# Posicionamento sobre Avaliação Pré-participação Cardiológica após a Covid-19: Orientações para Retorno à Prática de Exercícios Físicos e Esportes – 2020

**DOI:** 10.36660/abc.20210368

**Published:** 2021-06-08

**Authors:** Cléa Simone Sabino de Souza Colombo, Marcelo Bichels Leitão, Antônio Carlos Avanza, Serafim Ferreira Borges, Anderson Donelli da Silveira, Fabrício Braga, Ana Cristina Camarozano, Daniel Arkader Kopiler, José Kawazoe Lazzoli, Odilon Gariglio Alvarenga de Freitas, Gabriel Blacher Grossman, Mauricio Milani, Mauricio B. Nunes, Luiz Eduardo Fonteles Ritt, Carlos Alberto Cyrillo Sellera, Nabil Ghorayeb

**Affiliations:** 1 Faculdade de Medicina São Leopoldo Mandic CampinasSP Brasil Faculdade de Medicina São Leopoldo Mandic – Campinas, SP – Brasil; 2 Sportscardio Clínica Cardiológica ValinhosSP Brasil Sportscardio Clínica Cardiológica – Valinhos, SP – Brasil; 3 CEFIT – Centro de Estudos de Fisiologia do Exercício e Treinamento CuritibaPR Brasil CEFIT – Centro de Estudos de Fisiologia do Exercício e Treinamento, Curitiba, PR – Brasil; 4 Universidade Vila Velha ES Brasil Universidade Vila Velha, ES – Brasil; 5 Clínica Centrocor VitóriaES Brasil Clínica Centrocor, Vitória, ES – Brasil; 6 Clube de Regatas do Flamengo Rio de JaneiroRJ Brasil Clube de Regatas do Flamengo, Rio de Janeiro, RJ – Brasil; 7 Instituto Estadual de Cardiologia Aloysio de Castro Rio de JaneiroRJ Brasil Instituto Estadual de Cardiologia Aloysio de Castro, Rio de Janeiro, RJ – Brasil; 8 Imagecor Medicina Diagnóstica e do Exercício Rio de JaneiroRJ Brasil Imagecor Medicina Diagnóstica e do Exercício, Rio de Janeiro, RJ – Brasil; 9 Conselho Federal de Medicina Câmara técnica de Medicina Desportiva Rio de JaneiroRJ Brasil Conselho Federal de Medicina, Câmara técnica de Medicina Desportiva, Rio de Janeiro, RJ – Brasil; 10 Hospital de Clínicas de Porto Alegre Porto AlegreRS Brasil Hospital de Clínicas de Porto Alegre, Porto Alegre, RS – Brasil; 11 Universidade Federal do Rio Grande do Sul Porto AlegreRS Brasil Universidade Federal do Rio Grande do Sul, Porto Alegre, RS – Brasil; 12 Laboratório de Performance Humana Rio de JaneiroRJ Brasil Laboratório de Performance Humana, Rio de Janeiro, RJ – Brasil; 13 Casa de Saúde São José Rio de JaneiroRJ Brasil Casa de Saúde São José, Rio de Janeiro, RJ – Brasil; 14 Confederação Brasileira de Triathlon Rio de JaneiroRJ Brasil Confederação Brasileira de Triathlon, Rio de Janeiro, RJ – Brasil; 15 Universidade Federal do Paraná CuritibaPR Brasil Universidade Federal do Paraná, Curitiba, PR – Brasil; 16 Instituto Nacional de Cardiologia Rio de JaneiroRJ Brasil Instituto Nacional de Cardiologia (INC), Rio de Janeiro, RJ – Brasil; 17 Confederação Pan-Americana de Medicina do Esporte Brasil Confederação Pan-Americana de Medicina do Esporte; 18 Instituto Biomédico da Universidade Federal Fluminense NiteróiRJ Brasil Instituto Biomédico da Universidade Federal Fluminense (UFF), Niterói, RJ – Brasil; 19 Hospital Santa Teresa/ACSC PetrópolisRJ Brasil Hospital Santa Teresa/ACSC, Petrópolis, RJ – Brasil; 20 Confederação Pan-Americana de Medicina do Esporte Brasil Confederação Pan-Americana de Medicina do Esporte (COPAMEDE); 21 Federação Internacional de Medicina do Esporte Brasil Federação Internacional de Medicina do Esporte (FIMS); 22 Comissão de Autorização para Uso Terapêutico (CAUT) da Autoridade Brasileira de Controle de Dopagem Brasil Comissão de Autorização para Uso Terapêutico (CAUT) da Autoridade Brasileira de Controle de Dopagem (ABCD); 23 Minascor Centro Médico Belo HorizonteMG Brasil Minascor Centro Médico, Belo Horizonte, MG – Brasil; 24 Hospital Moinhos de Vento Porto AlegreRS Brasil Hospital Moinhos de Vento, Porto Alegre, RS – Brasil; 25 Clínica Cardionuclear Porto AlegreRS Brasil Clínica Cardionuclear, Porto Alegre, RS – Brasil; 26 Fitcordis Medicina do Exercício BrasíliaDF Brasil Fitcordis Medicina do Exercício, Brasília, DF – Brasil; 27 Hospital Português SalvadorBA Brasil Hospital Português, Salvador, BA – Brasil; 28 Hospital Cárdio Pulmonar SalvadorBA Brasil Hospital Cárdio Pulmonar, Salvador, BA – Brasil; 29 Escola Bahiana de Medicina e Saúde Pública SalvadorBA Brasil Escola Bahiana de Medicina e Saúde Pública, – Salvador, BA – Brasil; 30 Santa Casa de Santos SantosSP Brasil Santa Casa de Santos, Santos, SP – Brasil; 31 Universidade Metropolitana de Santos SantosSP Brasil Universidade Metropolitana de Santos, Santos, SP – Brasil; 32 Instituto Dante Pazzanese de Cardiologia São PauloSP Brasil Instituto Dante Pazzanese de Cardiologia, São Paulo, SP – Brasil; 33 Hospital do Coração São PauloSP Brasil Hospital do Coração (HCor), São Paulo, SP – Brasil

**Table:** Posicionamento sobre Avaliação Pré-participação Cardiológica após a Covid-19: Orientações para Retorno à Prática de Exercícios Físicos e Esportes – 2020

O relatório abaixo lista as declarações de interesse conforme relatadas à SBC pelos especialistas durante o período de desenvolvimento deste posicionamento, 2020.	
Especialista	Tipo de relacionamento com a indústria
Ana Cristina Camarozano	Nada a ser declarado
Anderson Donelli da Silveira	Nada a ser declarado
Antônio Carlos Avanza Junior	Nada a ser declarado
Carlos Alberto Cyrillo Sellera	Nada a ser declarado
Cléa Simone Sabino de Souza Colombo	Nada a ser declarado
Daniel Arkader Kopiler	Nada a ser declarado
Fabrício Braga	Nada a ser declarado
Gabriel Blacher Grossman	Nada a ser declarado
José Kawazoe Lazzoli	Nada a ser declarado
Luiz Eduardo Fonteles Ritt	Declaração financeira A - Pagamento de qualquer espécie e desde que economicamente apreciáveis, feitos a (i) você, (ii) ao seu cônjuge/companheiro ou a qualquer outro membro que resida com você, (iii) a qualquer pessoa jurídica em que qualquer destes seja controlador, sócio, acionista ou participante, de forma direta ou indireta, recebimento por palestras, aulas, atuação como proctor de treinamentos, remunerações, honorários pagos por participações em conselhos consultivos, de investigadores, ou outros comitês, etc. provenientes da indústria farmacêutica, de órteses, próteses, equipamentos e implantes, brasileiras ou estrangeiras: – Pfizer: Amiloidose Outros relacionamentos Possui qualquer outro interesse (financeiro ou a qualquer outro título) que deva ser declarado tendo em vista o cargo ocupado na sbc, ainda que não expressamente elencado anteriormente: – Consultor em projeto de pesquisa junto ao Cimatec, da empresa MDI Medical
Marcelo Bichels Leitão	Nada a ser declarado
Mauricio B. Nunes	Nada a ser declarado
Mauricio Milani	Nada a ser declarado
Odilon Gariglio Alvarenga de Freitas	Nada a ser declarado
Serafim Ferreira Borges	Nada a ser declarado
Nabil Ghorayeb	Nada a ser declarado

## 1. Introdução

Em 30 de janeiro de 2020, a Organização Mundial da Saúde (OMS) declarou que o surto da doença (COVID-19) causada pelo novo coronavírus (SARS-CoV-2) representava uma “Emergência de Saúde Pública de Importância Internacional” – o mais alto nível de alerta da organização, conforme previsto no Regulamento Sanitário Internacional. Comparado com o SARS-CoV que causou um surto de síndrome respiratória aguda grave (SARS) em 2003, o SARS-CoV-2 tem capacidade de transmissão mais alta. O rápido aumento de casos confirmados tornou a prevenção e o controle da COVID-19 extremamente importantes. O Ministério da Saúde recebeu a primeira notificação de um caso confirmado de COVID-19 no Brasil no dia 26 de fevereiro de 2020 e, em 11 de março de 2020, a doença foi caracterizada pela OMS como uma pandemia, o que trouxe uma necessidade emergencial de se buscar conhecimento, visando soluções o mais rápido possível, tanto para o tratamento quanto para a sua prevenção.^1^

A pandemia levou a medidas preventivas e restritivas em todo o mundo, e que foram diferentes nos países e continentes, dependendo da evolução da doença em cada região. Com o declínio das taxas de infecção, restrições menos rigorosas estão sendo implementadas para a prática de esportes e exercícios.

A COVID-19 tem sido associada a um número significativo de complicações cardiovasculares, atingindo cerca de 16% dos pacientes.^2^ No entanto, ainda não existem dados a longo prazo, muito menos em indivíduos ativos e atletas competitivos. Com base no conhecimento estabelecido sobre as miocardites virais em geral, é sabido que podem existir sequelas que afetam desde o desempenho físico desses indivíduos até a maior ocorrência de morte súbita (MS) durante o exercício, pois representam um substrato arritmogênico no miocárdio.^3^

O objetivo deste posicionamento consiste em alertar para o risco de comprometimento cardíaco e suas possíveis sequelas na COVID-19 e orientar sobre a necessidade de avaliação cardiológica após a doença antes do retorno à prática esportiva, propondo estratégias para a prevenção de MS através de uma avaliação pré-participação (APP) cardiológica direcionada.

## 2. Atividade Física e Pandemia

Frente à inexistência de tratamentos eficazes na eventualidade de uma infecção pelo SARS-CoV-2, a obtenção de medidas que reduzam o risco de contaminação é fundamental. Neste segmento, encontram-se as práticas amplamente difundidas de isolamento social, distanciamento, etiqueta respiratória, uso de máscaras e higienização frequente das mãos.

Contudo, busca-se identificar intervenções que melhorem a saúde da população, possibilitando uma diminuição do risco de infecção ou resposta clínica mais eficiente, de forma que o indivíduo apresente quadro clínico leve e com boa evolução, caso seja infectado pelo novo coronavírus. Modificações alimentares e suplementações com vitaminas estão entre as estratégias propostas. Entretanto, não há evidências consistentes em favor de qualquer uma dessas propostas profiláticas até o momento.^4^

Por ser uma doença nova, ainda não há dados disponíveis sobre como o exercício físico regular pode afetar a evolução da COVID-19.

Por outro lado, os benefícios da atividade física para a saúde estão bem estabelecidos. Indivíduos que fazem exercícios regulares, de modo geral, apresentam proteção contra viroses, com redução da incidência de infecções de vias aéreas superiores e melhor evolução clínica, com menos complicações.^5^ Essas evidências são documentadas em tipos distintos de infecções virais, inclusive em algumas causadas por rinovírus e alguns tipos de coronavírus.^6^ A prática regular de exercícios físicos em intensidade leve a moderada melhora a imunidade e pode colaborar como fator potencial para maior resistência a contrair a COVID-19 e a ter uma evolução mais favorável em uma eventual infecção.^7^^,^^8^

Dentre os benefícios mais importantes do exercício regular está a redução do risco cardiovascular através de diversos mecanismos, como redução da pressão arterial, dos níveis de lipídios sanguíneos, da glicemia, de marcadores inflamatórios e hemostáticos.^9^ A presença de doenças cardiovasculares e metabólicas está associada à maior mortalidade em pacientes infectados pelo SARS-CoV-2.

Outro fator relevante é a obesidade, que tem sido descrita como fator de risco importante para a gravidade da evolução da COVID-19, principalmente nos mais jovens. Estudos demonstram que os pacientes com índice de massa corporal (IMC) >30 kg/m^2^ evoluem mais frequentemente para ventilação mecânica invasiva, podendo ser um fator associado ao maior risco de óbito.^10^^–^^13^

O período de quarentena, com a imposição do confinamento das pessoas, tem ocasionado aumento da compulsão alimentar e do sedentarismo, contribuindo para o aumento da obesidade e para o descontrole de doenças como hipertensão e diabetes melito.

Outro aspecto que tem sido documentado desde o início da pandemia da COVID-19 é o aumento na incidência de distúrbios psicológicos em decorrência do confinamento doméstico. Foram reportadas elevadas taxas de ansiedade e depressão em indivíduos mantidos em quarentena devido à pandemia, e tem se discutido um possível aumento de suicídios.^14^^,^^15^ Assim como para a obesidade, também há dados consistentes na literatura documentando os efeitos da atividade física regular na redução de depressão, ansiedade e outros distúrbios da saúde mental.^16^

Portanto, é necessário buscar continuamente a otimização no tratamento dessas doenças, e o exercício físico tem papel essencial nesse controle. Dessa maneira, recomenda-se adotar e manter um estilo de vida ativo com o objetivo de melhorar a saúde e bem-estar em diversos aspectos, incluindo redução de risco cardiovascular, metabólico e melhor equilíbrio mental.

Apesar das restrições impostas pelo risco de contaminação pelo coronavírus, primordialmente, devemos estimular os indivíduos a se manterem fisicamente ativos, seja em exercícios em casa ou ao ar livre, respeitando as normas de higiene e distanciamento locais.

## 3. COVID-19

Os indivíduos acometidos pela COVID-19 apresentam sintomatologia muito variável, sendo que a maioria desenvolve quadro clínico leve a moderado, geralmente com sintomas gripais como tosse seca, dor de garganta, cefaleia e febre, tendo sido descritos também diarreia, *rush* cutâneo, perda de olfato e paladar. Uma proporção pequena dos doentes evolui com quadro mais grave, podendo apresentar falta de ar, dor torácica e perda de movimentos, necessitando hospitalização e suporte intensivo.^17^

A progressão da doença ao longo do tempo é dividida em três fases patológicas: uma fase inicial de infecção, uma fase pulmonar e uma grave fase de hiperinflamação. A fase inicial da infecção é caracterizada por infiltração e replicação viral. A doença progride para a fase pulmonar, caracterizada por comprometimento respiratório e alteração em exames de imagem pulmonar. A resposta inflamatória exagerada, impulsionada pela imunidade do hospedeiro, define a fase de hiperinflamação. Marcadores inflamatórios estão elevados neste estágio, e danos nos órgãos secundários se tornam aparentes.^18^^,^^19^

Embora as manifestações clínicas da COVID-19 sejam dominadas por sintomas respiratórios, alguns pacientes apresentam comprometimento cardiovascular grave.^20^ Além disso, alguns pacientes com doenças cardiovasculares (DCV) subjacentes podem ter aumento do risco de morte.^21^ Portanto, entender os danos causados pelo SARS-CoV-2 no sistema cardiovascular e os mecanismos subjacentes são da maior importância para que o tratamento desses pacientes possa ser oportuno e efetivo, com redução da mortalidade e de complicações tardias.

### 3.1. COVID-19 e Coração

Com base em dados de países como a China, onde a pandemia se originou, e de outros países com grande número de casos de COVID-19, como os EUA e a Itália, bem como metanálise sobre a doença, a lesão cardíaca parece ser uma característica proeminente da doença, ocorrendo em 20% a 30% dos pacientes hospitalizados e contribuindo para até 40% das mortes.^22^ Foram descritas complicações cardiovasculares, como lesão miocárdica (20% dos casos), arritmias (16%), miocardite (10%), além de insuficiência cardíaca congestiva (ICC) e choque (até 5% dos casos).^23^^,^^24^ Em um estudo que avaliou 138 pacientes internados por COVID-19, 16,7% desenvolveram arritmia e 7,2% apresentaram lesão cardíaca aguda (anormalidades eletrocardiográficas ou ecocardiográficas), sendo que quase 12% dos pacientes sem DCV conhecida previamente apresentaram níveis elevados de troponina T ultrassensível (Tnt) ou parada cardíaca durante a hospitalização.^25^^,^^26^ Notavelmente, a Tnt foi superior ao percentil 99, limite de referência em 46% dos não sobreviventes, em oposição a 1% dos sobreviventes.^27^ Seu aumento associou-se com outros biomarcadores inflamatórios (dímero D, ferritina, interleucina-6, lactato desidrogenase), aumentando a possibilidade de que isso reflita uma “tempestade de citocinas” ou linfo-histiocitose hemofagocítica secundária, mais do que lesão miocárdica isolada.^27^ Não se sabe se esse fenômeno é a principal causa de miocardite fulminante, se a resposta é puramente relacionada com inflamação, autoimunidade ou combinação de ambos, como observado em outros tipos de miocardite viral.^28^

Por outro lado, há relatos de pacientes que apresentam sintomas cardíacos predominantes que sugerem um padrão diferente, como cardiomiopatia por estresse e síndrome coronariana aguda, em que a fisiopatologia não está clara, mas que pode estar relacionada a um estado pró-trombótico associado à doença, como em indivíduos que apresentaram embolia pulmonar e acidente vascular cerebral (AVC).^15^^,^^24^^,^^29^^–^^31^ A fisiopatologia exata nos quadros graves de COVID-19 ainda é obscura e acredita-se que a lesão cardíaca pode resultar por mecanismos diretos ou indiretos (Quadro 1).^21^^,^^29^^,^^32^

**Quadro 1:**
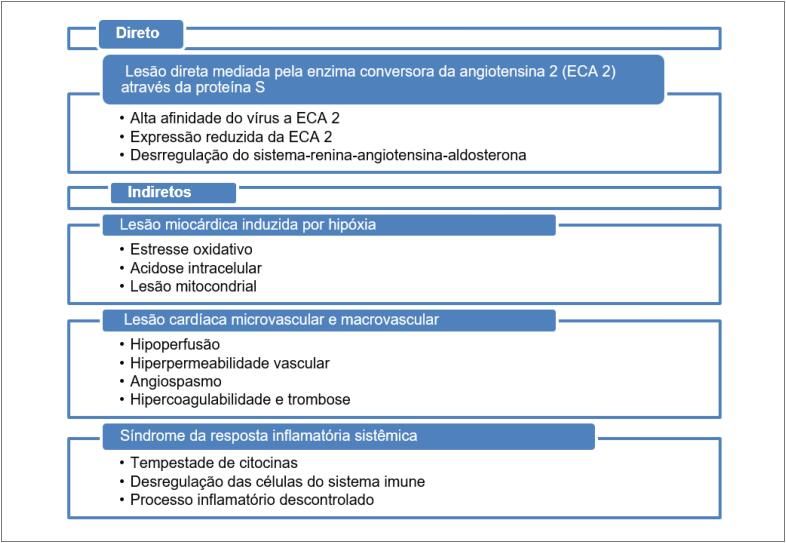
Mecanismos propostos para lesão cardíaca na COVID-19.

O envolvimento do coração com outras apresentações, tais como choque cardiogênico e insuficiência cardíaca, provavelmente teria o mesmo mecanismo fisiopatológico implicado.

## 4. Avaliação Pré-participação Esportiva

A APP cardiológica é a principal ferramenta para a prevenção de MS no esporte. A Diretriz em Cardiologia do Esporte e Exercício da Sociedade Brasileira de Cardiologia e da Sociedade de Medicina do Exercício e Esporte recomenda que todo indivíduo passe por uma avaliação médica antes de iniciar a prática de exercícios.^33^ Considerando-se que a maioria das pessoas parou ou reduziu seu treinamento físico durante a pandemia, é recomendável que, antes de retomá-lo, sejam submetidas à nova APP.

É de conhecimento geral que exercícios vigorosos podem levar à MS em indivíduos considerados suscetíveis, ou seja, que apresentam doença cardíaca subjacente geralmente não diagnosticada.^34^ Habitualmente, a APP tem o objetivo de identificar estes indivíduos, buscando as chamadas doenças cardiovasculares genéticas, relativamente incomuns, mas que representam as principais causas de MS no esporte, como cardiomiopatia hipertrófica, displasia arritmogênica ventricular, origem anômala de coronárias, aneurisma de aorta relacionado com síndrome de Marfan, síndrome do QT longo, síndrome de Brugada, entre outras. Entre as doenças adquiridas que podem levar à MS, destacam-se a doença arterial coronariana obstrutiva e a miocardite, esta principalmente em jovens. No contexto atual, atenção especial deve ser dada à possibilidade de agressão ao miocárdio e pericárdio pelo SARS-CoV-2. Como a COVID-19 é uma nova doença e o conhecimento a seu respeito ainda é limitado, deve ser feita uma avaliação criteriosa com o objetivo de afastar a presença e/ou sequela de miopericardite, mesmo nos indivíduos assintomáticos que testaram positivo.

Sendo assim, recomendamos que todos aqueles que tiveram COVID-19, assintomáticos ou não, passem por avaliação médica, preferencialmente cardiológica, incluindo pelo menos anamnese, exame físico e ECG de repouso de 12 derivações. Como o maior risco de MS está relacionado com maior intensidade do exercício, as recomendações de exames complementares na APP são diferentes conforme a prática esportiva. Neste documento, fizemos uma subdivisão do grupo chamado “esportista” em recreativos e competitivos, pois consideramos que há um número crescente de indivíduos que competem de forma amadora e sem vínculo profissional, mas que estão expostos a alto volume e intensidade de treino, aproximando-se dos chamados “atletas”. A definição desses grupos e de alguns conceitos importantes para sua compreensão encontram-se nos Quadros 2 e 3.^33^^,^^35^

**Quadro 2:**
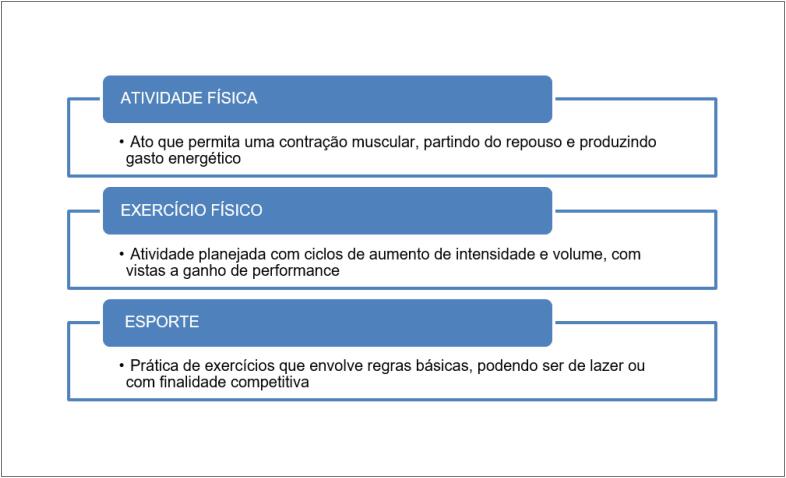
Conceitos de movimento. Fonte: Pescatello L et al.^35^

**Quadro 3:**
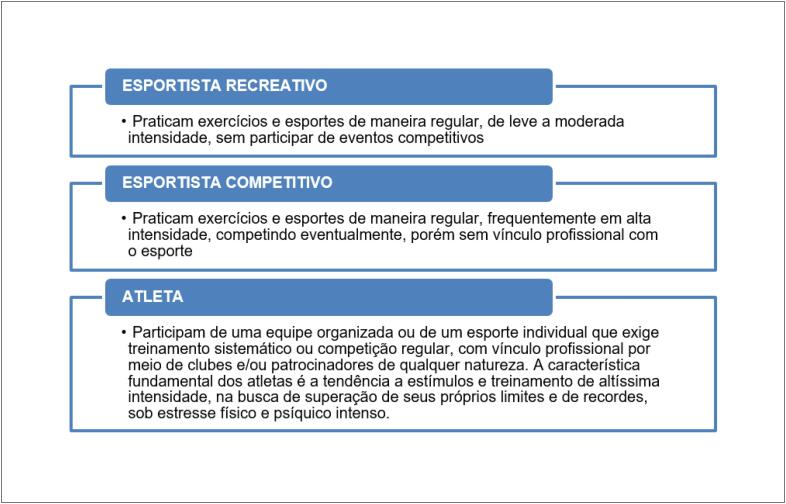
Definição dos grupos de praticantes de atividades físicas e esportes. Modifidado de: Ghorayeb N et al.^33^

**Figura 1:**
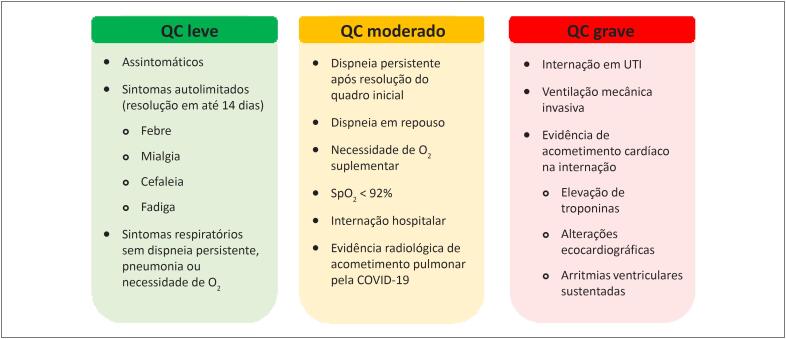
Definição de indivíduos com quadro clínico (QC) leve, moderado e grave de COVID-19. Adaptado de: Siddiqi HK & Mehra MR.^36^

Da mesma maneira, a indicação dos exames também pode variar de acordo com a gravidade do quadro clínico da doença. De acordo com a classificação atual proposta na literatura para os estágios da COVID-19, os doentes podem ser divididos em quadro clínico leve, moderado e grave, conforme o histórico apresentado (Figura 1).^36^

Devido à escassez de informações sobre a COVID-19 em crianças e adolescentes, e por apresentarem características diferentes, este documento não abordará esse grupo, sendo as recomendações sugeridas voltadas para a população em idade adulta.

Como critério de testagem positiva para a COVID-19, consideramos a existência prévia de exame de RT-PCR (identificação do RNA do vírus) associado a sintomas suspeitos ou de sorologia (identificação de IgG) positivos para SARS-CoV-2.^37^ Ainda não está bem estabelecido o significado da manutenção de exame de RT-PCR positivo em indivíduos assintomáticos após a resolução do quadro clínico. Dados recentes demonstraram que alguns indivíduos podem apresentar resquícios do RNA do SARS-CoV-2 até 12 semanas após o quadro infeccioso, porém sem replicação viral, não apresentando potencial de infecção.^38^ Sendo assim, não há indicação para se repetir o exame de RT-PCR após 3 a 4 dias da resolução dos sintomas, não sendo necessária a documentação de RT-PCR negativa para o término da quarentena, nem para o retorno à prática esportiva, sendo o critério de liberação baseado em dados clínicos.

Deve-se ressaltar que indivíduos em fase aguda da doença e/ou sintomáticos não podem reiniciar a prática de atividades físicas. Portanto, a APP deve ser realizada, no mínimo, após 14 dias do diagnóstico nos assintomáticos, ou 14 dias após a resolução do quadro clínico naqueles sintomáticos.

### 4.1. Exames Complementares

#### 4.1.1. Eletrocardiograma de 12 Derivações

O ECG de 12 derivações em repouso é recomendado na APP de esportistas e atletas em nossa Diretriz Brasileira de Cardiologia do Esporte, com o objetivo de identificar possíveis alterações que se correlacionem às doenças incipientes previamente citadas como causas mais comuns de MS.^39^ Particularmente nos indivíduos após a COVID-19, devemos estar atentos às alterações que podem estar relacionadas com pericardite ou miocardite. As mais comuns são:

- Alterações do segmento ST (geralmente depressão do segmento ST);
- Inversão da onda T;
- Distúrbios da condução, principalmente bloqueio completo do ramo esquerdo e bloqueio atrioventricular avançado;
- Arritmias supraventriculares e ventriculares complexas.^40^

Um estudo italiano em pacientes hospitalizados por COVID-19 associada à pneumonia demonstrou que 26% deles apresentaram novas alterações eletrocardiográficas até 51 dias (média de 20 a 30 dias) após o início dos sintomas, quando comparadas com o ECG de admissão. Os achados mais frequentes foram síndrome braditaquicardia (2%), fibrilação atrial (6%) e alterações ST persistentes (14%), sendo que em 38% desses pacientes foram identificados níveis alterados de Tnt associado. As alterações não se correlacionaram com a gravidade do quadro pulmonar, aparecendo às vezes na véspera da alta hospitalar e após novo teste de RT-PCR negativo.^41^

É importante ressaltar que indivíduos bem treinados e atletas geralmente apresentam um padrão eletrocardiográfico diferente da população geral, devido às adaptações fisiológicas cardíacas secundárias ao exercício. Portanto, a sua interpretação deve seguir as atuais “Recomendações internacionais para interpretação do ECG do atleta”, e ser preferencialmente realizada por cardiologista com experiência na área de esporte ou médico do esporte com experiência em cardiologia.^42^ Além disso, é de extrema utilidade a comparação do ECG pós-COVID-19 com um ECG anterior do esportista ou atleta, devendo ser considerado suspeito e passível de investigação adicional o surgimento de quaisquer novas alterações.

#### 4.1.2. Troponina T Ultrassensível

A troponina T ultrassensível (Tnt) é um importante marcador de lesão miocárdica, sendo a sua dosagem utilizada para auxílio diagnóstico em determinadas cardiopatias. A associação de elevação de seus níveis com alterações sugestivas de miocardite na ressonância magnética cardíaca (RMC) já está bem estabelecida e é há muito tempo conhecida. Apesar de a Tnt elevada durante a internação de pacientes com COVID-19 ter se mostrado um importante marcador prognóstico, ainda não temos uma correlação direta estabelecida entre esses dois achados nessa doença.^43^^–^^46^

Dados iniciais sobre pacientes em fase subaguda de COVID-19 com alterações compatíveis com miocardite na RMC demostraram elevação significativa dos níveis de Tnt (>9,3pg/mL), mas é interessante observar que 71% dos pacientes recuperados de COVID-19 apresentaram níveis de Tnt “detectável” (>3,0pg/mL).^47^ Até o presente momento, esta é a melhor informação que se tem de uma eventual associação de Tnt elevada com miocardite na COVID-19.

Sendo assim, consideramos que a dosagem ambulatorial de Tnt também na fase subaguda da doença pode ser uma ferramenta importante não só na estratificação de risco, como no rastreio de pacientes que deverão realizar RMC para melhor investigação diagnóstica.

#### 4.1.3. Teste Ergométrico

A realização do teste ergométrico (TE) tem várias indicações no âmbito da prática esportiva, desde avaliação da capacidade funcional (CF) até a identificação precoce de doenças cardiovasculares, arritmias e prognóstico. Nos esportistas após COVID-19, destaca-se a importância da presença de alterações do segmento ST e de arritmias durante ou após o esforço, bem como a quantificação da CF atingida. Entretanto, no caso da CF, o teste cardiopulmonar de exercício (TCPE) seria preferencial para uma avaliação mais precisa. Do mesmo modo que no ECG de repouso, a comparação com exames anteriores do mesmo paciente é de grande importância na interpretação dos achados do TE.

#### 4.1.4. Teste Cardiopulmonar

O teste cardiopulmonar de exercício (TCPE) é o padrão-ouro na avaliação da capacidade funcional máxima pela medida direta do consumo de oxigênio. Apresenta vantagens importantes em relação ao TE convencional por realizar medida mais acurada da CF, fornecer medidas prognósticas de eficiência ventilatória, auxiliar no diagnóstico diferencial da dispneia e conter critérios objetivos de maximalidade.^48^ O TCPE, em muitos casos, é capaz de esclarecer o mecanismo fisiopatológico principal da limitação ao exercício, auxiliando no diagnóstico e na conduta terapêutica apropriada. É um exame importante na diferenciação da gênese da dispneia sugerindo limitação pulmonar, cardiovascular ou por descondicionamento físico, conforme seus resultados.

Pouco se sabe sobre o papel do TCPE em pacientes pós-infecção pelo novo coronavírus. Até o momento, não dispomos de estudos em pacientes pós-COVID-19 publicados. Em um estudo realizado em pacientes com SARS, de amostra pequena, 75% dos indivíduos apresentavam exame alterado, 43% por descondicionamento, 19% por limitação cardiovascular e 6% por limitação pulmonar.^49^

Muitos atletas estão retornando às suas atividades e estarão eventualmente menos condicionados. No atual contexto, em que há a possibilidade de atletas que contraíram a COVID-19, mesmo em sua forma leve, apresentarem complicações cardiorrespiratórias tardias, a disponibilidade de um método que ajude a diferenciar baixo condicionamento de ineficiência cardiorrespiratória pode auxiliar na tomada de conduta desses atletas.

O TCPE fornece diversas informações sobre a eficiência ventilatória, sendo que a mais utilizada em pacientes com ICC é a inclinação da razão entre a ventilação e a produção de CO_2_ (VE/VCO_2_ *slope*).^50^^,^^51^ Existem trabalhos mostrando que o VE/VCO_2_ *slope* em atletas não se modifica, mesmo quando há variações significativas na CF máxima.^52^^,^^53^

Por seu potencial papel prognóstico adicional, pela possibilidade de auxílio no diagnóstico diferencial de dispneia e pela disponibilização de informações de eficiência ventilatória independentes da CF máxima, recomendamos o TCPE, quando disponível, para todos os indivíduos pós COVID-19 com dispneia a esclarecer, formas moderadas ou graves da doença e para todos os atletas competitivos.

#### 4.1.5. Holter 24h

O exame de holter 24h é útil na identificação de arritmias, sintomáticas ou não, e estará indicado em casos específicos quando há suspeita de lesão miocárdica com sequelas. A presença de arritmias é um dos critérios de avaliação prognóstica e de elegibilidade para retorno à prática esportiva em pacientes com diagnóstico de miocardite.^54^

#### 4.1.6. Ecodopplercardiograma

A realização do ecodopplercardiograma (ECO) é de grande utilidade na prática esportiva, por avaliar dados a respeito da fisiologia adaptativa do coração do atleta. Sua indicação está na identificação de alterações estruturais cardíacas que muitas vezes são responsáveis por MS nesses indivíduos. Por isso, a aplicação do ECO na triagem de atletas de alta *performance* é de grande importância para prevenir desfechos trágicos, uma vez que o método tem alta sensibilidade e especificidade em identificar essas alterações.^55^

O protocolo de APP da Sociedade Europeia de Cardiologia enfatiza três pontos principais: a história pessoal e familiar, o exame clínico e o ECG.^56^ No entanto, algumas doenças estruturais como cardiomiopatia incipiente e origem anômala de artérias coronárias podem passar despercebidas ao exame clínico e ao eletrocardiograma, mas seriam identificadas ao ECO. É fundamental o conhecimento das características e dos valores de normalidade das medidas realizadas no ECO de atletas, que diferem da população geral, para uma interpretação adequada do exame.^57^

Particularmente, nos indivíduos após a COVID-19, devemos estar atentos às alterações cardíacas sugestivas de miopericardite. Essas alterações podem estar presentes mais frequentemente nos indivíduos que cursam com as formas moderadas ou graves da doença, mas, eventualmente, também naqueles que cursam com a forma leve e apresentam sintomas como dor torácica e palpitação ou sinais de dispneia e intolerância ao esforço. Nesses casos, o ECO torna-se fundamental antes do retorno ao exercício, para avaliar função cardíaca e possíveis alterações residuais.^58^ Se houver a possibilidade de comparação com ECO prévio, qualquer nova alteração deve ser considerada anormal. Entretanto, alterações na contratilidade global ou segmentar do ventrículo esquerdo (VE) ou ventrículo direito (VD) (fração de ejeção [FEVE] ≤ 50% ou TAPSE ≤ 17mm), dilatação de câmaras cardíacas, presença de trombos cavitários e derrame pericárdico são achados que podem estar relacionados com miopericardite.^57^^,^^59^

Além disso, a avaliação cardíaca através de novas tecnologias oferecidas pelo ECO, como o *strain* bidimensional (ou *speckle tracking*) longitudinal, que é um marcador sensível de deformidade miocárdica capaz de avaliar a contratilidade de modo objetivo, quantitativo e precoce, demonstra um padrão de alteração contrátil predominantemente basal do VE afetado por miocardite pós-COVID-19, diferentemente da miocardite convencional; o *strain* bidimensional longitudinal do VD demonstrou ser capaz de prever maior mortalidade nos indivíduos acometidos pela COVID-19, estratificando aqueles de maior risco e de menor sobrevida, quando o *strain* do VD torna-se ≤ 20,5%, sendo esta uma análise que também é importante ser realizada e pode ser de grande auxílio, quando disponível.^60^^,^^61^ O ótimo valor de *cut-off* na análise funcional do VD foi de –23%, com sensibilidade de 94,4% e especificidade de 64,7%, sendo um parâmetro superior ao TAPSE no valor prognóstico.^56^ Por fim, o ECO deve checar se há dilatação do VD, especialmente ao “corte” apical de quatro câmaras, considerando o diâmetro diastólico basal do VD maior que 41mm, ou se a relação diâmetro VD/VE está ≥0,9. A hipocinesia/acinesia da parede livre do VD e a regurgitação tricúspide são mais prevalentes na vigência de dilatação desta câmara, e esses achados estão presentes em um terço dos pacientes mecanicamente ventilados ou naqueles com tromboembolismo pulmonar. O mecanismo de dilatação do VD ainda não está completamente esclarecido e parece ser multifatorial, incluindo evento trombótico, hipoxemia, vasoconstrição e dano viral direto, mas a presença de dilatação do VD está fortemente associada com mortalidade hospitalar.^62^

#### 4.1.7. Ressonância Magnética Cardíaca

A RMC tem se destacado como um método importante de avaliação de lesão miocárdica. A associação das técnicas de mapeamento T1 e T2 e de investigação de realce tardio após a injeção de gadolínio propicia a identificação de sinais de edema, inflamação e fibrose miocárdica, bem como a diferenciação entre etiologia isquêmica ou não. Em pacientes com suspeita de miocardite, é considerada o padrão-ouro para diagnóstico não invasivo.^63^

Apesar de percentual significativo dos pacientes hospitalizados com COVID-19 ter apresentado elevação dos níveis de Tnt, a utilização de RMC para investigação de miocardite na fase aguda foi restrita devido ao risco de contaminação de *staff*. Entretanto, dados iniciais com RMC após a recuperação clínica dos pacientes sugerem que a lesão miocárdica pode persistir fora da fase aguda e independente da gravidade da manifestação clínica da doença na fase aguda.

Um estudo alemão realizou RMC em 100 indivíduos com pelo menos 15 dias de resolução dos sintomas de COVID-19 (média de 71 dias) e RT-PCR negativo, e observou que 78% dos pacientes apresentaram achados anormais, 71% tinham níveis de Tnt detectável e 5% com elevação significativa (acima do percentil 99). Entre os pacientes estudados, apenas 33% haviam necessitado de internação hospitalar, sendo que 18% foram assintomáticos.^47^

Desta forma, acreditamos que a RMC tenha papel importante na investigação complementar durante a APP de determinados esportistas e atletas. Entretanto, devemos considerar que ainda é um exame de acesso limitado e de alto custo, nem sempre acessível a nossa população.

As situações em que sugerimos a realização da RMC em indivíduos após a fase aguda da COVID-19 estão descritas no Quadro 4.

**Quadro 4:**
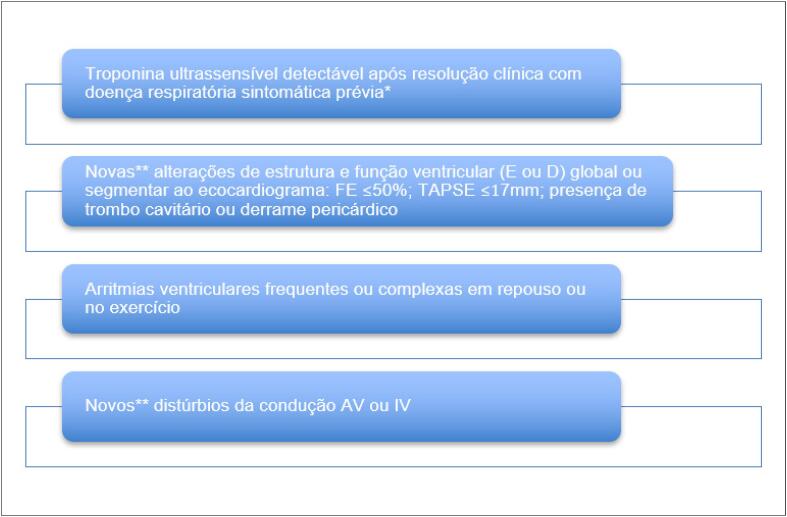
Situações em que há recomendação de realização de ressonância magnética cardíaca.

* Que não possam ser atribuídos a outras causas; **Associados ao início das doenças ou que não haja certeza da sua presença antes da COVID-19.

*AV: atrioventricular; D: direito; E: esquerdo; FE: fração de ejeção; IV: intraventricular; TAPSE: excursão sistólica do anel tricúspide.*

## 5. Recomendações para APP em Esportistas Recreativos

A APP é fundamental para segurança dos indivíduos que praticam exercícios. Os esportistas recreativos correspondem a um percentual significativo da população e pertencem às mais diversas faixas etárias. Com essa nova realidade que vivemos, é necessária uma adaptação da APP aos esportistas contaminados com COVID-19.

### 5.1. Grupo com Quadro Clínico Leve ou Assintomático

Os indivíduos que apresentaram quadro clínico leve, após permanecerem 14 dias assintomáticos, devem passar por uma avaliação médica com anamnese, exame físico e ECG, devendo ser considerada a possibilidade de realização de dosagem de Tnt. Com base nas informações que temos até o momento, devemos assumir que a presença de qualquer nível detectável de Tnt é um achado anormal, que pode estar associado a uma lesão miopericárdica tardia, identificada fora da fase aguda da doença.

Se a avaliação for normal, pelo menos após 14 dias de resolução dos sintomas, esses indivíduos estão liberados para reiniciar atividades físicas leves, com progressão gradual de intensidade e treinamento.

Caso seja detectada alguma alteração, deve-se progredir na investigação, seguindo a sequência sugerida para quadro clínico moderado.

### 5.2. Grupo com Quadro Clínico Moderado

Os indivíduos que apresentaram quadro clínico moderado devem realizar, após pelo menos 14 dias da resolução da doença, além da anamnese, exame físico e ECG, ECO, Tnt e TE ou TCPE, se disponível. Preferencialmente, o ECO deve ser realizado primeiro, pois, caso haja sinais de disfunção ventricular ou pericardite, o esforço máximo estaria contraindicado no momento. Se os exames forem normais, as atividades físicas podem ser retomadas de forma gradual, com monitoramento dos sintomas. Como a evolução da COVID-19 ainda não é bem conhecida, e aparentemente algumas alterações no coração podem ocorrer de forma tardia ou até mesmo se perpetuar, sugerimos uma reavaliação médica em 60 dias.

Caso apareçam anormalidades, deve-se prosseguir a investigação com RMC e, havendo sinais sugestivos de miocardite, realização de holter de 24h e demais exames necessários, conforme as diretrizes para orientação em casos de miocardite.^54^

### 5.3. Grupo com Quadro Clínico Grave

Os indivíduos que tiveram quadro clínico grave de COVID-19 devem realizar protocolo semelhante aos de quadro clínico moderado; no entanto, é importante considerar a realização de RMC mesmo se todos os exames forem normais. Há descrição de casos que não apresentam alterações ao ECG ou ECO, mas apresentaram áreas de realce tardio na RMC quando submetidos à investigação adicional, especialmente naqueles que cursaram com quadro clínico grave da doença, em que o acometimento cardíaco é relativamente frequente. Se houver alteração nos exames, devem seguir com investigação conforme orientação nas diretrizes de miocardite, incluindo holter de 24h, com retorno à prática esportiva conforme os critérios específicos de elegibilidade. O mesmo valendo quando for identificada alguma arritmia na avaliação inicial ou na prova funcional.

**Figura 2:**
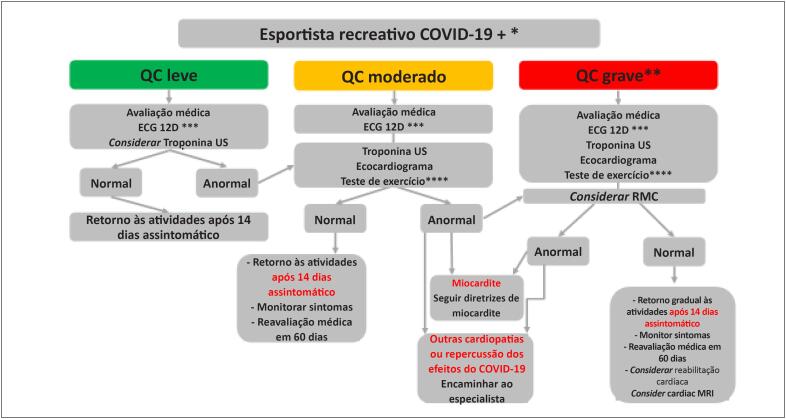
Fluxograma de avaliação para esportistas recreativos

No final da avaliação, se estiver tudo normal, deve-se aguardar duas semanas sem sintomas para reiniciar atividades físicas, monitorando reaparecimento de sintomas após o retorno. Neste grupo, pode haver a necessidade de retorno mais gradativo e até reabilitação cardíaca, dependendo do grau de comprometimento cardíaco na fase aguda e suas possíveis sequelas (Figura 2).

* RT-PCR ou teste imunológico; ** Aguardar no mínimo 3 meses para aqueles com diagnóstico de miocardite na fase aguda; *** Seguir critérios de avaliação do ECG do atleta/comparar com exame prévio; **** Se disponível, realizar teste cardiopulmonar de exercício. ECG: eletrocardiograma; RMC: ressonância magnética cardíaca.

## 6. Recomendações para APP em Esportistas Competitivos e Atletas

Neste grupo, encontram-se indivíduos que habitualmente treinam em alta intensidade, incluindo atletas profissionais, sendo que, no momento, alguns já têm reiniciado seus treinamentos e até competições. Com o retorno de alguns clubes de futebol em algumas regiões do nosso país, a testagem sorológica tem sido feita de rotina como triagem, mesmo naqueles sem histórico de doença prévia. Há relatos isolados de indivíduos que se recuperam da COVID-19 e desenvolvem complicações cardiovasculares mesmo na ausência de doença cardiovascular subjacente e também MS em indivíduos positivos para COVID-19 não hospitalizados, mesmo com sintomas leves.^64^ Portanto, devemos submetê-los a protocolos rigorosos para uma volta segura aos esportes competitivos. Vários modelos de protocolo têm sido propostos recentemente, nacional e internacionalmente, com o intuito de se chegar a um consenso de qual seria a melhor abordagem para APP desses atletas, e serviram de referência para a proposta deste documento.^65^^–^^67^

Nosso objetivo é orientar o retorno com segurança aos atletas e *staff* médico/técnico, na tentativa da reintegração e proteção de sequelas que tornariam este atleta inelegível para continuar em sua carreira competitiva ou mesmo sob o risco de sofrer MS.

Em atletas competitivos, manter as suas habilidades e aptidão, retomando treinamento intenso para atingir o nível exigido para a competição em curto período de tempo, gera maior desgaste físico e emocional, com grande ansiedade.^66^ O suporte médico adequado é importante para minimizar o impacto dessas condições.

Neste grupo, devido ao histórico de treinamento intenso, pode ser difícil diferenciar as alterações habituais do ECG do atleta de outras patologias. Portanto, é de extrema relevância a comparação com ECG prévio do atleta, bem como avaliação complementar com outros exames, mesmo nos casos mais leves.

### 6.1. Grupo com Quadro Clínico Leve ou Assintomático

Após permanecerem pelo menos 14 dias assintomáticos, todos devem ser submetidos à avaliação médica com anamnese, exame físico, ECG e dosagem de Tnt. Se não houver anormalidades, recomenda-se a realização de TE ou TCPE, se disponível. Sendo o teste normal, considera-se o atleta apto para retomar exercícios de baixos volume e intensidade, progredindo conforme protocolo funcional da modalidade. Exames de laboratórios protocolares de cada instituição em modelo de início de temporada podem ser adicionados.

Caso haja anormalidades, a investigação deve prosseguir como naqueles que apresentaram quadro clínico moderado, antes do retorno às atividades físicas.

### 6.2. Grupo com Quadro Clínico Moderado Risco

A avaliação dos atletas com quadro clínico classificado como moderado deve incluir anamnese, exame físico, ECG, Tnt, ECO e TE, preferencialmente TCPE (sempre, no mínimo, 14 dias após a resolução da doença). Havendo alteração dos níveis de Tnt, mesmo em vigência de ECO normal, sugerimos complementar investigação com RMC. Caso haja sinais sugestivos de miocardite, a avaliação deve continuar conforme as orientações vigentes para a doença, que inclui holter 24h e outros exames para estratificação de risco e elegibilidade para retorno às atividades físicas.^54^

Se a avaliação for normal, considera-se o atleta apto para retomar exercícios de baixos volume e intensidade 14 dias após resolução do quadro clínico, com retorno gradual à intensidade maior e treinamento específico, devendo ser monitorado o aparecimento de sintomas. Sugere-se uma reavaliação médica após 30 dias da APP inicial, visto que podem ocorrer manifestações cardíacas de forma tardia e novas alterações eletrocardiográficas em indivíduos que tiveram COVID-19 com pneumonia e necessitaram de hospitalização (classificados neste grupo).^41^

### 6.3. Grupo com Quadro Clínico Grave

Para atletas que apresentaram quadro clínico classificado como grave, sugerimos APP abrangente, incluindo RMC mesmo com todos os demais exames normais. Em caso de alterações suspeitas de miocardite, esses atletas seguem as recomendações para investigação, estratificação de risco e elegibilidade já estabelecidas para a doença.^54^

É importante ressaltar que os indivíduos que tiveram diagnóstico de miocardite confirmado durante a fase aguda da doença devem se manter afastados de atividades físicas por um período mínimo de 3 meses antes de serem submetidos a esta APP inicial, seguindo as recomendações previamente citadas.

**Figura 3:**
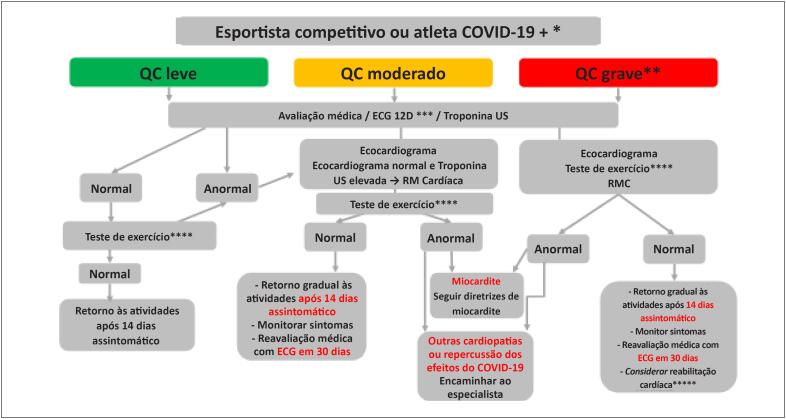
Fluxograma de avaliação para esportistas competitivos e atletas

Se todos os exames durante a APP estiverem normais, considera-se o atleta apto para o retorno às atividades no mínimo 14 dias após resolução da doença, com retorno gradual à intensidade maior e treinamento específico, com monitoramento cuidadoso do aparecimento de sintomas ou alteração de *performance*. Sugere-se uma reavaliação médica com ECG após 30 dias da APP inicial. Mesmo entre os atletas, pode haver indivíduos que necessitem ser encaminhados para a reabilitação cardíaca antes do retorno às atividades habituais, considerando a magnitude das lesões e sequelas miocárdicas possíveis neste grupo de alta gravidade (Figura 3).

* RT-PCR ou teste imunológico; ** Aguardar no mínimo 3 meses para aqueles com diagnóstico de miocardite na fase aguda; *** Seguir critérios de avaliação do ECG do atleta/comparar com exame prévio; **** Se disponível, realizar teste cardiopulmonar de exercício; ***** Ver texto. ECG: eletrocardiograma; RMC: ressonância magnética cardíaca.

## 7. Conclusão

Apesar de ainda não sabermos o real significado dos achados relatados até o momento, a possibilidade de comprometimento cardíaco como sequela da COVID-19, especialmente a miocardite, deve ser considerada e investigada antes do retorno à prática esportiva, visto que pode representar um substrato arritmogênico durante o esforço, aumentando o risco de morte súbita em esportistas e atletas.

Consideramos essencial a realização de uma avaliação pré-participação cardiológica após a resolução do quadro clínico, que inclui anamnese, exame físico e ECG para todos, podendo ser necessária a complementação com dosagem de Tnt e realização de TE ou TCPE, ECO e RMC, especialmente em atletas e esportistas competitivos. Indivíduos com diagnóstico de miocardite estabelecido na fase aguda da doença devem esperar, no mínimo, 3 meses para realizar APP e avaliar a possibilidade de retomar exercícios.

Além disso, sugerimos que os indivíduos que cursaram com COVID-19 e se recuperaram sem sequelas aparentes, especialmente os atletas, além da APP inicial, devam ser avaliados a médio e longo prazo para a elegibilidade plena para a prática de esportes competitivos e de alta intensidade, visto o pouco conhecimento ainda sobre a evolução tardia desta doença.

Finalizando, ficam as sugestões aqui colocadas com as informações que temos até o momento, mesmo que sem grandes evidências, por se tratar de doença ainda em descobertas e aprendizado. Ressaltamos que tais recomendações podem ser temporárias e sofrer alterações à luz do conhecimento futuro sobre a COVID-19.
