# Supplementary material for: Posicionamento sobre Avaliação Pré-participação Cardiológica após a Covid-19: Orientações para Retorno à Prática de Exercícios Físicos e Esportes – 2020
Source: Arq Bras Cardiol. 2021 Jun 8;116(6):1213–26. [Article in Portuguese] doi: 10.36660/abc.20210368 (PMC8288531; doi:10.36660/abc.20210368)
Supplement: Translation

# Translation: Position Statement on Post-COVID-19 Cardiovascular Preparticipation Screening: Guidance for Returning to Physical Exercise and Sports – 2020

**Authors:** Cléa Simone Sabino de Souza Colombo, Marcelo Bichels Leitão, Antônio Carlos Avanza, Serafim Ferreira Borges, Anderson Donelli da Silveira, Fabrício Braga, Ana Cristina Camarozano, Daniel Arkader Kopiler, José Kawazoe Lazzoli, Odilon Gariglio Alvarenga de Freitas, Gabriel Blacher Grossman, Mauricio Milani, Mauricio B. Nunes, Luiz Eduardo Fonteles Ritt, Carlos Alberto Cyrillo Sellera, Nabil Ghorayeb

**Affiliations:** 1 CampinasSP Brasil Faculdade de Medicina São Leopoldo Mandic – Campinas, SP – Brasil; 2 ValinhosSP Brasil Sportscardio Clínica Cardiológica – Valinhos, SP – Brasil; 3 CuritibaPR Brasil CEFIT – Centro de Estudos de Fisiologia do Exercício e Treinamento, Curitiba, PR – Brasil; 4 ES Brasil Universidade Vila Velha, ES – Brasil; 5 VitóriaES Brasil Clínica Centrocor, Vitória, ES – Brasil; 6 Rio de JaneiroRJ Brasil Clube de Regatas do Flamengo, Rio de Janeiro, RJ – Brasil; 7 Rio de JaneiroRJ Brasil Instituto Estadual de Cardiologia Aloysio de Castro, Rio de Janeiro, RJ – Brasil; 8 Rio de JaneiroRJ Brasil Imagecor Medicina Diagnóstica e do Exercício, Rio de Janeiro, RJ – Brasil; 9 Rio de JaneiroRJ Brasil Conselho Federal de Medicina, Câmara técnica de Medicina Desportiva, Rio de Janeiro, RJ – Brasil; 10 Porto AlegreRS Brasil Hospital de Clínicas de Porto Alegre, Porto Alegre, RS – Brasil; 11 Porto AlegreRS Brasil Universidade Federal do Rio Grande do Sul, Porto Alegre, RS – Brasil; 12 Rio de JaneiroRJ Brasil Laboratório de Performance Humana, Rio de Janeiro, RJ – Brasil; 13 Rio de JaneiroRJ Brasil Casa de Saúde São José, Rio de Janeiro, RJ – Brasil; 14 Rio de JaneiroRJ Brasil Confederação Brasileira de Triathlon, Rio de Janeiro, RJ – Brasil; 15 CuritibaPR Brasil Universidade Federal do Paraná, Curitiba, PR – Brasil; 16 Rio de JaneiroRJ Brasil Instituto Nacional de Cardiologia (INC), Rio de Janeiro, RJ – Brasil; 17 Brasil Confederação Pan-Americana de Medicina do Esporte; 18 NiteróiRJ Brasil Instituto Biomédico da Universidade Federal Fluminense (UFF), Niterói, RJ – Brasil; 19 PetrópolisRJ Brasil Hospital Santa Teresa/ACSC, Petrópolis, RJ – Brasil; 20 Brasil Confederação Pan-Americana de Medicina do Esporte (COPAMEDE); 21 Brasil Federação Internacional de Medicina do Esporte (FIMS); 22 Brasil Comissão de Autorização para Uso Terapêutico (CAUT) da Autoridade Brasileira de Controle de Dopagem (ABCD); 23 Belo HorizonteMG Brasil Minascor Centro Médico, Belo Horizonte, MG – Brasil; 24 Porto AlegreRS Brasil Hospital Moinhos de Vento, Porto Alegre, RS – Brasil; 25 Porto AlegreRS Brasil Clínica Cardionuclear, Porto Alegre, RS – Brasil; 26 BrasíliaDF Brasil Fitcordis Medicina do Exercício, Brasília, DF – Brasil; 27 SalvadorBA Brasil Hospital Português, Salvador, BA – Brasil; 28 SalvadorBA Brasil Hospital Cárdio Pulmonar, Salvador, BA – Brasil; 29 SalvadorBA Brasil Escola Bahiana de Medicina e Saúde Pública, – Salvador, BA – Brasil; 30 SantosSP Brasil Santa Casa de Santos, Santos, SP – Brasil; 31 SantosSP Brasil Universidade Metropolitana de Santos, Santos, SP – Brasil; 32 São PauloSP Brasil Instituto Dante Pazzanese de Cardiologia, São Paulo, SP – Brasil; 33 São PauloSP Brasil Hospital do Coração (HCor), São Paulo, SP – Brasil

**Table:** Position Statement on Post-COVID-19 Cardiovascular Preparticipation Screening: Guidance for Returning to Physical Exercise and Sports – 2020

The report below lists declarations of interest as reported to the SBC by the experts during the period of the development of these statement, 2020.	
Expert	Type of relationship with industry
Ana Cristina Camarozano	Nothing to be declared
Anderson Donelli da Silveira	Nothing to be declared
Antônio Carlos Avanza Junior	Nothing to be declared
Carlos Alberto Cyrillo Sellera	Nothing to be declared
Cléa Simone Sabino de Souza Colombo	Nothing to be declared
Daniel Arkader Kopiler	Nothing to be declared
Fabrício Braga	Nothing to be declared
Gabriel Blacher Grossman	Nothing to be declared
José Kawazoe Lazzoli	Nothing to be declared
Luiz Eduardo Fonteles Ritt	Financial declaration A - Economically relevant payments of any kind made to (i) you, (ii) your spouse/partner or any other person living with you, (iii) any legal person in which any of these is either a direct or indirect controlling owner, business partner, shareholder or participant; any payments received for lectures, lessons, training instruction, compensation, fees paid for participation in advisory boards, investigative boards or other committees, etc. from the brazilian or international pharmaceutical, orthosis, prosthesis, equipment and implants industry: – Pfizer: Amiloidose Other relationships Any other interest — financial or other — that should be declared considering the position taken in sbc that has not been expressly listed above: – Consultor em projeto de pesquisa junto ao Cimatec, da empresa MDI Medical
Marcelo Bichels Leitão	Nothing to be declared
Mauricio B. Nunes	Nothing to be declared
Mauricio Milani	Nothing to be declared
Odilon Gariglio Alvarenga de Freitas	Nothing to be declared
Serafim Ferreira Borges	Nothing to be declared
Nabil Ghorayeb	Nothing to be declared

## 1. Introduction

On January 30, 2020, the World Health Organization (WHO) stated that the outbreak of coronavirus disease 2019 (COVID-19) caused by the new coronavirus (SARS-CoV-2) constituted a “public health emergency of international concern” — the highest-level alert issued by the organization, as provided for in the International Health Regulations. Compared to SARS-CoV, which caused an outbreak of severe acute respiratory syndrome (SARS) in 2003, SARS-CoV-2 has greater transmission potential. The rapid increase in confirmed cases has made COVID-19 prevention and control extremely important. The Brazilian Ministry of Health received the first report of a confirmed case of COVID-19 in Brazil on February 26, 2020; on March 11, 2020, WHO declared the disease a pandemic, leading to an urgent need to seek knowledge and solutions as quickly as possible for both treatment and prevention.^1^

The pandemic led to preventive and restrictive measures worldwide, which were different across countries and continents depending on COVID-19 outcomes in each region. As infection rates decline, less strict restrictions are being implemented for sports and exercise.

COVID-19 has been associated with a significant number of cardiovascular complications, reaching approximately 16% of patients.^2^ However, there is a lack of long-term data, especially on active individuals and competitive athletes. Based on the established knowledge about common viral myocarditis, it is known that there may be sequelae affecting physical performance and leading to greater occurrence of sudden death (SCD) during exercise, as they constitute an arrhythmogenic substrate in the myocardium.^3^

This position statement aims to warn against the risk of cardiac impairment and its possible sequelae in patients with COVID-19 as well as to provide guidance on the need for post-disease cardiological evaluation before returning to sports, including proposed strategies for SCD prevention using targeted cardiovascular preparticipation screening (PPS).

## 2. Physical Activity and the Pandemic

In view of the lack of effective treatments in the event of a SARS-CoV-2 infection, obtaining measures that reduce the risk of contamination is essential. These include the widespread practices of social isolation, distancing, respiratory etiquette, wearing masks, and frequent hand hygiene.

However, interventions that improve the health of the population and thus allow a reduction in the risk of infection or a more efficient clinical response are also required, so that individuals have mild symptoms and good outcomes if infected with the new coronavirus. Dietary changes and vitamin supplements are among the proposed strategies. However, there is no consistent evidence in favor of any of those prophylactic measures to date.^4^

As this is a new disease, there is a lack of data on how regular exercise may affect the course of COVID-19.

Conversely, the health benefits of physical activity are well established. Overall, individuals who exercise regularly are protected against viruses, with reduced incidence of upper airway infections and better clinical outcomes with fewer complications.^5^ Such evidence has been documented in different types of viral infections, including some caused by rhinovirus and some types of coronavirus.^6^ Regular light-to-moderate intensity exercise improves immunity and may contribute as a potential factor for having greater resistance to develop COVID-19 and for having more favorable outcomes in an occasional infection.^7^^,^^8^

The most important benefits of regular exercise include reduced cardiovascular risk through several mechanisms, such as reduced levels of blood pressure, blood lipids, blood glucose, and inflammatory and hemostatic markers.^9^ The presence of cardiovascular and metabolic diseases is associated with higher mortality in SARS-CoV-2-infected patients.

Another relevant factor is obesity, which has been described as an important risk factor for the severity of COVID-19 symptoms, especially in the young. Studies show that patients with body mass index (BMI) > 30 kg/m^2^ require invasive mechanical ventilation more frequently, which may be a factor associated with a higher risk of death.^10^^–^^13^

The quarantine period, with the imposition of people’s confinement, has caused an increase in binge eating and sedentary lifestyle, contributing to an increased prevalence of obesity and a loss of control over diseases such as hypertension and diabetes mellitus.

Another feature that has been documented since the beginning of the COVID-19 pandemic is the increased incidence of psychological disorders due to home confinement. High rates of anxiety and depression have been reported in quarantined individuals because of the pandemic, and a possible increase in suicides has been discussed.^14^^,^^15^ As is the case with obesity, there is also consistent literature documenting the effects of regular physical activity on reducing depression, anxiety, and other mental health disorders.^16^

Therefore, treatment of those diseases must be continuously optimized, and exercise plays an essential role in control measures. Thus, adopting and maintaining an active lifestyle is recommended to improve several aspects of health and well-being, including reduced cardiovascular and metabolic risk and improved mental balance.

Despite the restrictions imposed by the risk of coronavirus contamination, we should primarily encourage individuals to remain physically active, regardless of whether they exercise at home or outdoors, respecting local hygiene and distancing rules.

## 3. COVID-19

Individuals with COVID-19 have a wide range of symptoms, with the majority showing mild-to-moderate manifestations, especially flu-like symptoms such as dry cough, sore throat, headache, and fever, as well as diarrhea, skin rash, and loss of smell and taste. A small proportion of patients develops more severe symptoms and may experience shortness of breath, chest pain, and loss of movements, requiring hospitalization and intensive support.^17^

The progression of the disease over time is divided into three pathological stages: an early infection stage, a pulmonary stage, and a severe hyperinflammation stage. The early infection stage is characterized by viral infiltration and replication. The disease then progresses to the pulmonary stage, characterized by respiratory impairment and changes in pulmonary imaging tests. An exaggerated inflammatory response, driven by host immunity, defines the hyperinflammation stage. Inflammatory markers are elevated at this stage, and damage to secondary organs becomes apparent.^18^^,^^19^

Although clinical manifestations of COVID-19 are dominated by respiratory symptoms, some patients have severe cardiovascular impairment.^20^ Some patients with underlying cardiovascular disease (CVD) may also be at an increased risk of death.^21^ Therefore, understanding the damage caused by SARS-CoV-2 to the cardiovascular system and the underlying mechanisms is of utmost importance so that the treatment of those patients can be timely and effective, with reduced mortality and late complications.

### 3.1. COVID-19 and the Heart

Based on data from countries such as China, where the pandemic began, from other countries with a large number of COVID-19 cases, such as the United States (US) and Italy, and from a meta-analysis on the disease, cardiac injury appears to be a prominent feature, affecting 20% to 30% of hospitalized patients and contributing to 40% of deaths.^22^ Cardiovascular complications have been described, including myocardial injury (20% of cases), arrhythmia (16%), myocarditis (10%), and congestive heart failure (CHF) and shock (up to 5%).^23^^,^^24^ In a study evaluating 138 patients hospitalized with COVID-19, 16.7% developed arrhythmia and 7.2% had acute cardiac injury (electrocardiographic or echocardiographic abnormalities). Almost 12% of patients without any previously known CVD had elevated levels of high-sensitivity troponin T (TnT) or cardiac arrest during hospitalization.^25^^,^^26^ Notably, TnT was above the 99th percentile upper reference limit in 46% of nonsurvivors as opposed to 1% of survivors.^27^ Its elevation was associated with other inflammatory biomarkers (D-dimer, ferritin, interleukin-6, lactate dehydrogenase), increasing the possibility that this reflects a “cytokine storm” or secondary hemophagocytic lymphohistiocytosis rather than myocardial injury alone.^27^ It is unknown whether this phenomenon is the main cause of fulminant myocarditis and whether the response is purely related to inflammation, autoimmunity, or a combination of both, as seen in other types of viral myocarditis.^28^

Conversely, there are reports of patients with predominant cardiac symptoms that suggest a different pattern, such as stress cardiomyopathy and acute coronary syndrome, in which pathophysiology is unclear but may be related to a prothrombotic state associated with the disease, as seen in individuals who had pulmonary embolism and stroke.^15^^,^^24^^,^^29^^–^^31^ The exact pathophysiology in severe cases of COVID-19 remains unclear, and cardiac injury is believed to result from direct or indirect mechanisms (Chart 1).^21^^,^^29^^,^^32^

**Chart 1:** Proposed mechanisms for cardiac injury in COVID-19

Cardiac involvement with other presentations, such as cardiogenic shock and heart failure, would probably entail the same pathophysiological mechanism.

## 4. Cardiovascular Preparticipation Screening

Cardiovascular PPS is the main tool for SCD prevention in sports. The Guidelines for Exercise and Sports Cardiology, published by the Brazilian Society of Cardiology and the Brazilian Society of Exercise and Sports Medicine, recommend that all individuals undergo a medical evaluation before beginning to exercise.^33^ Considering that most people stopped or reduced their physical training during the pandemic, it is recommended that, before resuming it, they undergo a new PPS evaluation.

It is well known that vigorous exercise may lead to SCD in susceptible individuals, ie, those who have underlying, usually undiagnosed heart disease.^34^ Overall, PPS aims to identify such individuals, searching for so-called genetic cardiovascular diseases, which are relatively uncommon but represent the main causes of SCD in sports, such as hypertrophic cardiomyopathy, arrhythmogenic ventricular dysplasia, anomalous origin of coronary arteries, aortic aneurysm related to Marfan syndrome, long QT syndrome, Brugada syndrome, among others. Acquired diseases that may lead to SCD include obstructive coronary artery disease and myocarditis, especially in the young. In the current context, special attention should be given to a possible aggression to the myocardium and pericardium by SARS-CoV-2. Because COVID-19 is a new disease and relevant knowledge remains limited, a careful evaluation should be conducted to rule out the presence and/or sequela of myopericarditis, even in asymptomatic individuals who tested positive.

Therefore, we recommend that all those who had COVID-19, asymptomatic or not, undergo a medical assessment, including at least medical history and physical examination, and resting 12-lead electrocardiogram (ECG). As a higher risk of SCD is related to greater exercise intensity, the recommendations for additional PPS tests are different according to sports practice. In this document, we divided the amateur athletes into recreational and competitive, as we believe that there is an increasing number of individuals who compete as amateurs and have no professional relationships but who are exposed to high volume and intensity training, similar to the so-called professional “athletes.” How those groups are defined and some important concepts for understanding them are shown in Charts 2 and 3.^33^^,^^35^

**Chart 2:** Concepts of movement. Source: Pescatello L et al.^35^

**Chart 3:** Definition of groups engaging in physical activities and sports. Modified from: Ghorayeb N et al.^33^

Likewise, indication for tests may also vary according to the severity of COVID-19 ilness. In the current classification proposal for COVID-19 stages, patients can be divided into mild, moderate and severe, according to the clinical presentation (Figure 1).^36^

**Figure 1:** Definition of mild, moderate and severe clinical presentation of COVID-19 ilness. Adapted from: Siddiqi HK & Mehra MR.^36^

Because information about COVID-19 in children and adolescents is limited and because they have different characteristics, this document will not address this group. The suggested recommendations are focused on the adult population.

As a criterion for positive COVID-19 testing, we considered the existence of previous reverse-transcription polymerase chain reaction (RT-PCR) (viral RNA identification) associated with suspicious symptoms or serology (IgG identification) positive for SARS-CoV-2.^37^ The reason why some asymptomatic individuals remain positive on RT-PCR after clinical resolution is not well established. Recent data have shown that some individuals may have traces of SARS-CoV-2 RNA for up to 12 weeks after the infection but no viral replication, with no potential for infection.^38^ Hence, repeating the RT-PCR test 3 to 4 days after symptom resolution is not indicated, and there is no need for negative RT-PCR documentation to end quarantine or to return to sports, as clearance criteria are based on clinical data.

It is worth noting that individuals who are in the acute phase and/or symptomatic cannot resume physical activity. Therefore, PPS should be conducted at least 14 days after diagnosis in asymptomatic patients or 14 days after clinical resolution in symptomatic patients.

### 4.1. Additional Tests

#### 4.1.1. 12-Lead Electrocardiogram

Resting 12-lead ECG is recommended in PPS of amateur and professional athletes in the Brazilian Guidelines for Sports Cardiology to identify possible changes that correlate with incipient diseases that were previously mentioned as the most common causes of SCD.^39^ Particularly in individuals post COVID-19, we must be aware of changes that may be related to pericarditis or myocarditis. The most common ones are:

- ST-segment changes (usually ST-segment depression);
- T-wave inversion;
- Conduction abnormalities, especially complete left bundle branch block and atrioventricular blocks;
- Complex supraventricular and ventricular arrhythmias.^40^

An Italian study of patients hospitalized with COVID-19 associated with pneumonia showed that 26% had new electrocardiographic changes within 51 days (mean, 20 to 30 days) of symptom onset when compared to admission ECG. The most frequent findings were bradycardia (2%), atrial fibrillation (6%), and persistent ST changes (14%); in 38% of patients, increased levels of associated TnT were identified. The changes did not correlate with severity of pulmonary symptoms, sometimes appearing on the eve of hospital discharge and after a new negative RT-PCR test.^41^

It is important to point out that well-trained individuals and athletes usually have an electrocardiographic pattern different from that of the general population due to physiological cardiac adaptations secondary to exercise. Therefore, interpretation should be done preferably by a cardiologist with experience in sports or a sports medicine physician with experience in cardiology, following the current Ïnternational recommendations for electrocardiographic interpretation in athletes.^42^ Additionally, comparing a post-COVID-19 ECG with a previous ECG of the athlete is extremely useful, and any new changes should be considered suspect and subject to further investigation.

#### 4.1.2. High-Sensitivity Troponin T

High-sensitivity TnT is an important marker of myocardial injury, and its assay is used to help in the diagnosis of some heart diseases. The association of elevated levels with changes suggestive of myocarditis on cardiac magnetic resonance imaging (MRI) is well established and has long been known. Although elevated TnT during hospitalization of patients with COVID-19 has proved to be an important prognostic marker, a direct correlation between those two findings has not been established in this disease.^43^^–^^46^

Initial data on patients at the subacute stage of COVID-19 presenting cardiac MRI changes compatible with myocarditis showed a significant increase in TnT levels (> 9.3 pg/mL), but interestingly, 71% of recovered patients had “detectable” TnT levels (> 3.0 pg/mL).^47^ To date, this is the best available information on a possible association of elevated TnT with myocarditis in COVID-19.

Hence, we consider that outpatient TnT levels dosage also in the subacute stage of disease can be an important tool not only for stratifying risk but also for screening patients who should undergo cardiac MRI for further diagnostic investigation.

#### 4.1.3. Exercise Testing

Exercise testing (ExT) has several indications in the sports field, including assessment of functional capacity (FC) and early identification and prognosis of cardiovascular diseases and arrhythmias. In athletes post COVID-19, it is important to note the occurrence of ST segment changes and arrhythmias during or after exercise, and FC assessment at the peak exercise, as well. However, in the case of FC, a cardiopulmonary exercise test (CPET) is preferable for a more accurate assessment. As in resting ECG, comparing new and previous tests of the same patient is of great importance in the interpretation of ExT findings.

#### 4.1.4. Cardiopulmonary Exercise Testing

CPET is the gold standard for assessment of maximal FC using direct measurement of oxygen uptake. It has important advantages over conventional ExT, including measuring FC more accurately, providing prognostic measures of ventilatory efficiency, assisting in differential diagnosis of dyspnea, and using objective criteria for maximality.^48^ CPET, in many cases, is able to elucidate the main pathophysiological mechanism of exercise limitation, helping in diagnosis and appropriate therapeutic approach. It is an important test for identifying the genesis of dyspnea suggesting pulmonary, cardiovascular, or physical deconditioning limitations, depending on results.

Little is known about the role of CPET in patients post new coronavirus infection. To date, there are no published studies on patients post COVID-19. In a study of a small sample of patients with SARS, 75% had abnormal tests — 43% due to deconditioning, 19% due to cardiovascular limitation, and 6% due to pulmonary limitation.^49^

Many athletes are returning to their activities and will occasionally be less conditioned. In the current context, as there is the possibility that athletes who contracted COVID-19, even in its mild presentation, have late cardiorespiratory complications, an available method that helps differentiate low conditioning from cardiorespiratory inefficiency can assist in the approach for those athletes.

CPET provides a range of information about ventilatory efficiency, and the relationship between minute ventilation and carbon dioxide production (VE/VCO_2_ slope) is the most used parameter in patients with CHF.^50^^,^^51^ There are studies showing that VE/VCO_2_ slope in athletes does not change, even when there are significant variations in maximal FC.^52^^,^^53^

Because of the potential additional prognostic role, the possibility of assisting in differential diagnosis of dyspnea, and the availability of information on ventilatory efficiency regardless of maximal FC, we recommend CPET, if available, for all individuals post COVID-19 with dyspnea who had severe or moderate clinical presentation and for all competitive athletes.

#### 4.1.5. 24-Hour Holter Monitoring

A 24-hour Holter test is useful for identifying arrhythmias, either symptomatic or not, and will be indicated for specific cases when myocardial injury with sequelae is suspected. The presence of arrhythmias is one of the criteria for prognostic evaluation and for eligibility to return to sports in patients diagnosed with myocarditis.^54^

#### 4.1.6. Echocardiography

Echocardiography (Echo) is particularly useful in sports for evaluating data regarding adaptive physiology of athlete’s heart. Echo is indicated for identification of cardiac structural changes that are often responsible for SCD in those individuals. Therefore, the use of Echo for screening high-performance athletes is of great importance to prevent tragic outcomes, since the method has high sensitivity and specificity for identifying those changes.^55^

The European Society of Cardiology PPS protocol highlights three main points: personal and family history, clinical examination, and ECG.^56^ However, some structural diseases such as incipient cardiomyopathy and anomalous origin of coronary arteries may be missed by clinical examination and ECG but will be identified on Echo. It is essential to know the characteristics and normal values of athletes’ Echo measures, which differ from those of the general population, for adequate test interpretation.^57^

Particularly in individuals post COVID-19, we must be aware of cardiac changes suggestive of myopericarditis. Those changes may be more frequently present in individuals who have moderate or severe illness but, occasionally, also in those who have mild illness presenting symptoms such as chest pain and palpitation or signs of dyspnea and effort intolerance. In such cases, Echo becomes essential before returning to exercise to assess cardiac function and possible residual changes.^58^ If there is a possibility of comparing new and previous Echo tests, any new change should be considered abnormal. However, changes in global or segmental contractility of the left ventricle (LV) or right ventricle (RV) (ejection fraction [EF] ≤ 50% or tricuspid annular plane systolic excursion [TAPSE] ≤ 17 mm), dilation of cardiac chambers, presence of intracardiac thrombi, and pericardial effusion are findings that may be related to myopericarditis.^57^^,^^59^

Moreover, cardiac assessment using new Echo technologies, such as two-dimensional longitudinal strain (or speckle tracking), which is a sensitive marker of myocardial deformation capable of assessing contractility in an objective, quantitative, and early manner, demonstrates a pattern of predominantly basal contractile change in the LV affected by post-COVID-19 myocarditis, which is different from conventional myocarditis. Two-dimensional longitudinal strain of the RV was able to predict higher mortality in individuals with COVID-19, stratifying those at greater risk and shorter survival, when RV strain became ≤ 20.5%; thus, this is another important analysis that can be of great help when available.^60^^,^^61^ The optimal cut-off value in RV function analysis was –23%, with 94.4% sensitivity and 64.7% specificity, thus being a parameter superior to TAPSE in terms of prognostic value.^56^ Finally, Echo must check if there is RV dilation, especially on the apical 4-chamber view, considering a baseline RV diastolic diameter greater than 41 mm, or if RV/LV diameter ratio is ≥ 0.9. Hypokinesia/akinesia of the RV free wall and tricuspid regurgitation are more prevalent in the presence of chamber dilation, and they are found in one third of mechanically ventilated patients or in those with pulmonary thromboembolism. The RV dilation mechanism is not completely understood and appears to be multifactorial, including thrombotic event, hypoxemia, vasoconstriction, and direct viral damage, but the presence of RV dilation is strongly associated with hospital mortality.^62^

#### 4.1.7. Cardiac Magnetic Resonance Imaging

Cardiac MRI has emerged as an important method for assessing myocardial injury. The association of T1 and T2 mapping and late gadolinium enhancement provides the identification of signs of edema, inflammation, and myocardial fibrosis, as well as the differentiation between ischemic and nonischemic etiology. In patients with suspected myocarditis, cardiac MRI is the gold standard for noninvasive diagnosis.^63^

Although a significant percentage of patients hospitalized with COVID-19 showed increased TnT levels, the use of cardiac MRI for investigating myocarditis in the acute phase was limited because of the risk of staff contamination. However, initial cardiac MRI data on clinically recovered patients suggests that myocardial injury may persist after the acute phase and regardless of severity of clinical manifestations in the acute phase.

A German study used cardiac MRI in 100 participants with at least 15 days of COVID-19 symptom resolution (mean, 71 days) and negative RT-PCR tests, and reported that 78% of patients had abnormal findings, 71% had detectable TnT levels, and 5% had significant elevation (above the 99th percentile). Among the study patients, only 33% had required hospitalization and 18% had been asymptomatic.^47^

Therefore, we believe that cardiac MRI has an important role in additional PPS investigation of some athletes. However, we should consider that this is a test of limited access and high cost, not always accessible to our population.

Our indications for using cardiac MRI in individuals post acute phase of COVID-19 are described in Chart 4.

**Chart 4:** When we recommend using cardiac magnetic resonance imaging

* Which cannot be attributed to other causes; ** Associated with the onset of diseases or whose presence is uncertain before COVID-19. L: left; R: right; EF: ejection fraction; TAPSE: tricuspid annular plane systolic excursion; AV: atrioventricular; IV: intraventricular.

## 5. Recommendations for PPS in Recreational Amateur Athletes

PPS is essential for practicing exercise safely. Recreational amateur athletes account for a significant percentage of the population and belong to a wide range of age groups. In this new reality that we are living in, PPS must be adapted to amateur athletes contaminated with COVID-19.

### 5.1. Mild Clinical Presentation

Individuals who had mild ilness, after remaining asymptomatic for 14 days, should undergo a medical evaluation that includes medical history, physical examination, and ECG, and the possibility of measuring TnT should be considered. Based on the information we have to date, we should assume that the presence of any detectable TnT level is an abnormal finding, which may be associated with late myopericardial injury identified out of the acute phase.

If the evaluation is normal, individuals must wait at least 14 days after symptom resolution and then are free to resume light physical activities with gradual progression of intensity and training.

If any changes are detected, additional investigation should be made based on the suggested sequence for moderate ilness.

### 5.2. Moderate Clinical Presentation

Individuals who had moderate clinical presentation should undergo, at least 14 days after disease resolution, Echo, TnT, and ExT or CPET, if available, in addition to medical history, physical examination, and ECG. Preferably, Echo should be performed first because, if there are signs of ventricular dysfunction or pericarditis, maximal effort is contraindicated. If the tests are normal, physical activities can be resumed gradually, with monitoring of symptoms. As the course of COVID-19 remains not well known, and apparently some changes in the heart may occur late or even persist, we suggest a medical reevaluation in 60 days.

If abnormalities appear, investigation should continue with cardiac MRI and, in the case that myocarditis is suspected, 24-hour Holter monitoring and other required tests, according to the guidelines for cases of myocarditis.^54^

### 5.3. Severe Clinical Presentation

Individuals who had severe COVID-19 clinical presentation should undergo a protocol similar to that of the moderate ones; however, cardiac MRI should be considered even if all tests are normal. There have been descriptions of cases that had no changes on ECG or Echo but showed areas of late enhancement on cardiac MRI during additional investigation, especially in those who had severe ilness, in which cardiac involvement is relatively frequent. If there are changes in the tests, investigation should continue according to myocarditis guidelines, including a 24-hour Holter test, following the specific elegibility criteria to return to sports practice. The same applies to when an arrhythmia is identified on initial evaluation or functional test.

At the end of the evaluation, if results are all normal, individuals should wait two weeks without symptoms before resuming physical activities, and reappearance of symptoms should be monitored after the return. In this group, there may be a need for a more gradual return and even cardiac rehabilitation, depending on the degree of cardiac involvement in the acute phase and possible sequelae (Figure 2).

**Figure 2:** Flowchart for evaluation of recreational amateur athletes

* RT-PCR or immunologic test; ** Wait at least 3 months for those diagnosed with myocarditis in the acute phase; *** Follow athlete’s ECG evaluation criteria/compare with a previous test; **** If available, perform a cardiopulmonary exercise test. ECG: electrocardiogram; TnT: troponin T; MRI: magnetic resonance imaging.

## 6. Recommendations for PPS in Competitive Amateur Athletes and Professional Athetes

In this group, there are individuals who usually do high-intensity training, and, at the moment, some have already resumed training and even competitions. With the return of some football clubs in some Brazilian regions, serological testing has been routinely used for screening, even in those with no history of previous disease. There are isolated reports of individuals recovering from COVID-19 and developing cardiovascular complications even in the absence of underlying cardiovascular disease as well as nonhospitalized COVID-19-positive individuals having SCD, even with mild symptoms.^64^ Therefore, they must undergo strict protocols for a safe return to competitive sports. Several protocol models have recently been proposed, both nationally and internationally, in order to reach a consensus on what the best approach for athletes’ PPS is, and served as a reference for the proposal of this document.^65^^–^^67^

Our aim is to guide a safe return for athletes and medical/technical staff, in an attempt to reintegrate these athletes and protect them from sequelae that would make them ineligible to continue their competitive career or even put the athlete at risk of SCD.

In competitive athletes, maintaining their skills and fitness by resuming intense training to reach the level required for competition in a short period of time generates greater physical and emotional stress, with increased anxiety.^66^ Adequate medical support is important to minimize the impact of those conditions.

In this group of athletes, because of the history of intense training, it may be difficult to differentiate usual ECG changes from other diseases. Therefore, comparing new and previous ECGs and conducting additional tests, even in the mildest cases, are extremely important.

### 6.1. Mild Clinical Presentation

After remaining at least 14 days asymptomatic, all patients must undergo medical evaluation that includes medical history, physical examination, ECG, and TnT dosage. If there are no abnormalities, ExT or CPET are recommended, if available. If the test is normal, the athlete is considered fit to resume low volume and intensity exercises, progressing according to the functional protocol of each modality. Protocol laboratory examinations of each institution in an early season model can be added.

In case of abnormalities, the investigation should continue as in moderate ilness before they return to physical activities.

### 6.2. Moderate Clinical Presentation

The evaluation of athletes who had moderate clinical presentation should include medical history, physical examination, ECG, TnT, Echo, and ExT, preferably CPET (always at least 14 days after disease resolution). If there are changes in TnT levels, even when Echo is normal, we suggest additional investigation with cardiac MRI. If there are signs suggestive of myocarditis, the evaluation should continue according to current myocarditis guidelines, which includes a 24-hour Holter test and other tests for risk stratification and eligibility to return to physical activities.^54^

If the evaluation is normal, the athlete is considered fit to resume low volume and intensity exercises 14 days after symptom resolution, with gradual return to greater intensity and specific training, and appearance of symptoms should be monitored. A medical reevaluation is suggested after 30 days of the initial PPS, since late cardiac manifestations may occur and new electrocardiographic changes may appear in individuals who had COVID-19 associated with pneumonia requiring hospitalization (classified in this group).^41^

### 6.3. Severe Clinical Presentation

For athletes who were severe clinical presentation, we suggest a comprehensive PPS evaluation, including cardiac MRI even if all other tests are normal. In case of suspected myocarditis changes, these athletes should follow the established recommendations for investigation, risk stratification, and eligibility.^54^

It is worth noting that individuals who were diagnosed with confirmed myocarditis during the acute phase must stay away from physical activities for at least 3 months before undergoing an initial PPS evaluation, following the previously mentioned recommendations.

If all tests during PPS are normal, the athlete is considered fit to return to activities at least 14 days after disease resolution, with gradual return to greater intensity and specific training as well as careful monitoring for symptoms or changes in performance. A medical reevaluation with ECG is suggested 30 days after the initial PPS evaluation. Even among athletes, there may be those who need to be referred to cardiac rehabilitation before returning to their usual activities because of the magnitude of injuries and possible myocardial sequelae in this high-severity group (Figure 3).

**Figure 3:** Flowchart for evaluation of competitive: amateur and profissional athletes

* RT-PCR or immunologic test; ** Wait at least 3 months for those diagnosed with myocarditis in the acute phase; *** Follow athlete’s ECG evaluation criteria/compare with a previous test; **** If available, perform a cardiopulmonary exercise test; ***** See text. ECG: electrocardiogram; TnT: troponin T; MRI: magnetic resonance imaging.

## 7. Conclusion

Although we still do not know the real meaning of the findings reported to date, the possibility of cardiac involvement as a COVID-19 sequela, especially myocarditis, should be considered and investigated before individuals return to sports practice, since it may constitute an arrhythmogenic substrate during effort, thus increasing the risk of SCD in athletes.

We believe that cardiovascular PPS after full recovery from COVID-19 is very important, which includes medical history, physical examination, and ECG for all patients. Further investigation such as TnT dosage, ExT or CPET, ECHO and cardiac MRI may be necessary, especially in competitive amateur and professional athletes. Individuals diagnosed with myocarditis in the acute phase must wait at least 3 months before undergoing PPS and considering the possibility of resuming exercise.

Moreover, we suggest that individuals who had COVID-19 and recovered without apparent sequelae, especially athletes, in addition to undergoing an initial PPS evaluation, should be evaluated in the medium and long term for full eligibility to compete in high-intensity sports, given the limited amount of knowledge about the late course of the disease.

Finally, the suggestions made herein are based on the information we have to date, even if there is a lack of strong evidences, since learning and discovery are still emerging about this disease. We highlight that such recommendations may be temporary and may change in the light of future knowledge about COVID-19.
